# Women in Pharmacy

**Published:** 1915-09-04

**Authors:** 


### WOMEN IN PHARMACY.

To women pharmacists the war has brought an opportunity which is too valuable to neglect. Indeed, there is excellent evidence that they are making the most of the opportunity, for not long after the outbreak of war the leaders among them set to work to mobilise women pharmacists with the object of utilising their services to the best advantage of all concerned. Ever since the Insurance Act came into force there has been a scarcity of chemists' assistants, and this was greatly emphasised when the war broke out and so many young pharmacists joined the Forces in one capacity or another. The consequence was that qualified women were much sought after, not only as dispensers in public institutions, but as assistants in pharmacies. At the present time there are between two and three hundred women who possess the diploma of the Pharmaceutical Society, and the number of women students is growing each year. One has only to recall that the Jacob Bell Memorial Scholarship, the highest award available for women students, was this year won by a woman, and that the Fairchild Scholarship was awarded to a member of the same sex, to show the capabilities of women in this direction. In order to become a pharmacist it is necessary to pass a suitable preliminary examination in English, arithmetic, algebra, geometry, and two optional subjects, one of which ought, in the interests of the student, to be Latin. Then follows a pupilage of at least three years with a pharmacist and a course of instruction at one of the many schools of pharmacy. At the age of twenty-one years the candidate can enter for the qualifying examination, the passing of which gives her the legal right to dispense and sell poisons and to use the title of "pharmacist" or "chemist and druggist." In normal times about two-thirds of the qualified women are employed as dispensers in public institutions or in doctors' dispensaries, while a fair number, which is steadily increasing, are in business as chemists with pharmacies of their own. There is an Association of Women Pharmacists, with headquarters at Gordon Hall, Gordon Square, London, W.C., and the secretary would be very pleased to advise women who contemplate adopting pharmacy as a career.

